# Necrotizing Myositis in a Neutropenic Patient: The Use of Ultrasound in the Diagnosis of Atypical Presentations

**DOI:** 10.1155/2014/685263

**Published:** 2014-06-09

**Authors:** Maria Del Carmen Torrejón, Edgardo Celi, David Cancho, Ailie Knox, Cesar Henriquez-Camacho

**Affiliations:** ^1^Department of Trauma and Orthopedics, Hospital Universitario Fundacion Alcorcon, Calle Budapest 1, 28922 Madrid, Spain; ^2^Department of Surgery, Hospital Universitario Fundacion Alcorcon, Calle Budapest 1, 28922 Madrid, Spain; ^3^Department of Anesthesiology, Hospital Universitario Fundacion Alcorcon, Calle Budapest 1, 28922 Madrid, Spain; ^4^School of Medicine, University of Manchester, Stopford Building, Oxford Road, Manchester M13 9PT, UK; ^5^Infectious Diseases/Internal Medicine Unit, Hospital Universitario Fundacion Alcorcon, Calle Budapest 1, 28922 Madrid, Spain

## Abstract

We report a case of fatal necrotizing soft tissue infection (NSTI) due to *Clostridium perfringens* (CP) in a neutropenic patient with diabetes mellitus. As in many cases, by the time a diagnosis was made, the condition had rapidly progressed to its late stages, resulting in a fatal outcome. The emergency physician should be aware of NSTI as a complication when patients present with pain out of proportion to physical findings and/or signs of soft tissue compromise. Negative prognostic factors for survival are diabetes mellitus, immunosuppression, age, and toxic shock syndrome. A bedside ultrasound scan allows for rapid evaluation in time-sensitive critically ill patients and can promote prompt treatment without the need to delay for further imaging studies.

## 1. Clinical Case

**Figure 1:**
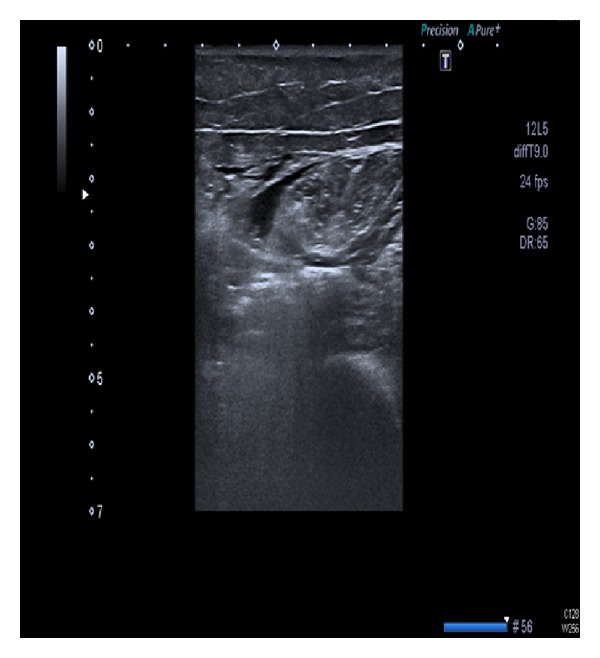
Ultrasound of thigh: edema and fluid bands around anterior thigh muscles.

**Figure 2:**
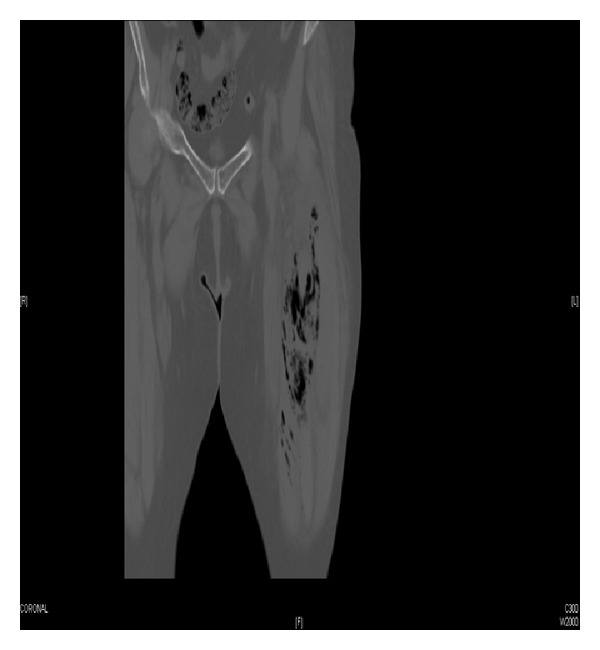
TC: edema and air on fascia between rectus femoris and vastus medialis.

A 76-year-old woman with a past medical history of diabetes mellitus, leukemia, and pancytopenia presented to the emergency department with a twelve-hour history of leg pain. On arrival, the patient was alert and afebrile with normal vital signs (temp 36°C, BP 114/68 mmHg, HR 80 bpm, and RR 14 breaths/min). Clinical examination was normal except for an intense pain in the left thigh, aggravated by movement, with no signs of edema or cellulitis. There was no evidence of crepitations or fluctuation on physical. Chest radiography and ECG were unremarkable. Initial blood tests revealed that leukocytes 860/mm^3^ (370 neutrophils), C-reactive protein 5, creatinine phosphokinase (CK) 360 U/L, d-dimer 1145 ng/mL, and biochemical analyses were normal. No abnormalities of the femur were detected on plain film radiography. Ultrasound exploration showed edema and fluid bands around the anterior thigh muscles with air-on-fascia between rectus femoris and vastus medialis (see Figure 1). A computed tomography (CT) scan confirmed the ultrasound findings (see Figure 2) and clindamycin with piperacilin/tazobactan was started. Urgent surgical debridement and excision of necrotic tissue were performed. Surgical findings revealed grey necrotic tissue, with lack of blood supply, and noncontracting muscle. 48 hours later further debridement of subcutaneous tissue and muscle was carried out. Within hours, the patient collapsed with hemolytic anemia and multiorganic failure and died shortly after. CP was isolated from both tissue and blood cultures.

## 2. Discussion

NSTI includes necrotizing forms of cellulitis, myositis, and fasciitis. These infections are characterised clinically by fulminant tissue destruction, systemic signs of toxicity, and high mortality. Necrotizing myositis (NM) (spontaneous gangrenous myositis) is relatively rare and is characterized by progressive and extensive destruction, leading to systemic toxicity, limb loss, and/or death. Even with surgery, mortality is high (20–40%) [1]. Clinical presentation can be initially nonspecific or asymptomatic until a significant deterioration culminates in sepsis or multiorganic failure [2]. In most cases, NSTI is polymicrobial. The most common pathogens are* Streptococcus*,* Clostridium spp.*,* Staphylococcus*,* Vibrio spp.*,* Aeromonas hydrophila,* and* Pasteurella spp.* [3].

NSTI can affect all populations; however, specific groups with a high proportion of intravenous drug use have been shown to present with outbreaks of NSTI [4]. A retrospective study evaluated risk factors such as diabetes mellitus, obesity, alcohol abuse, malnutrition, recent surgery, prior history of trauma, immunosuppression, malignancy, drug abuse, and chronic renal disease [5, 6]. Negative prognostic factors for survival were diabetes mellitus, immunosuppression, age, and toxic shock syndrome [7].

In the case of neutropenic patients the colon is thought to be the site of entry for nontraumatic “spontaneous” clostridial infections in both adults and children [8].

Patients with NSTI usually present with one or more of pain, swelling, and fever. This clinical triad is common in early necrotizing fasciitis, but during the early stages patients can have minimal cutaneous manifestations, making prompt diagnosis extremely difficult. Pain out of proportion to clinical findings during physical examination is the most consistent feature noted at the time of presentation, and this was true in the case of our patient. These symptoms may progress fairly quickly to the more typical signs of tense edema, ecchymosis, blisters/bullae, crepitus, and necrosis. Systemic findings include fever, tachycardia, hypotension, and shock. The presence of hemodynamic instability, crepitance, bullae with fluid, and skin necrosis, although helpful, are often late findings only present in a small percentage of patients. The presence of these signs however should trigger expeditious and aggressive surgical debridement [4].

In the diagnosis of NSRI, laboratory findings are generally nonspecific. Abnormalities may include leukocytosis, coagulopathy, and elevations in the serum creatine kinase (CK), lactate, and creatinine concentrations; but laboratory tests are of limited use in the setting of early infection. Laboratory risk indicator for necrotizing fasciitis (LRINEC) was found to have high sensitivity (93%) and high specificity (92%) in a Singaporean population [9], although subsequent data have demonstrated limited sensitivity [10].

With regard to imaging, plain-film radiography is a common modality used during investigation of a patient with suspected NSTI. Plain-film radiographs can help identify subcutaneous gas, however, are of limited use in early diagnosis as this finding is often associated with late phases of NSTI. Computed tomography (CT) scanning has a higher accuracy and can identify other possible causes of infection [9]. Bedside ultrasound allows for rapid evaluation in time-sensitive critically ill patients and thus can promote prompt treatment without having to delay for further radiography studies. Bedside ultrasound also has a role amongst pregnant patients who may not be able to undergo radiographic studies due to large amounts of fetal radiation exposure [11, 12].

In the case of our patient, the reported ultrasound showed thickened, distorted fascia with perifascial hypoechoic fluid collection and variable swelling of the subcutaneous tissues and muscle. These findings correlate well with histological results [13] and show signs of what we retrospectively know to be early muscle necrosis [14].

A high suspicion of NSTI should prompt urgent empirical antibiotic treatment and surgical evaluation. Once the diagnosis is confirmed, emergency surgical debridement is indicated [15]. Antibiotics such as penicillin, piperacillin/tazobactam, or carbapenems are used with clindamycin (this is bacteriostatic and inhibits* Streptococcus* superantigen [16]). Some experimental adjunct therapies (hyperbaric oxygen or intravenous immunoglobulins) have been reported in selected groups but the results are contradictory and controversial [17].

Our patient was affected by* Clostridium perfringens*, a Gram-positive, anaerobic bacterium, from the commensal flora of the digestive and female genital tract. It may be found in various clinical sources and is responsible for three distinctive histotoxic clostridial syndromes (gas gangrene, food poisoning, and enteritis necroticans). Clostridial myonecrosis is a devastating and rapidly progressive infection, due to the systemic effects of potent endotoxins produced by CP. In this case study the combination of immune suppression and rapid progression of the disease was determinant for the patient's poor prognosis.

For the emergency physician, in the case of a neutropenic patient who presents with limb pain which is out of proportion to physical findings without typical symptoms of NSTI, we recommend rapid assessment with bedside ultrasound (if available), laboratory studies, and CT scans of the affected limb. Prompt treatment with antibiotics and early surgical evaluation is crucial in the prevention of poor prognostic outcomes.

## References

[1] Anaya DA, Dellinger EP (2007). Necrotizing soft-tissue infection: diagnosis and management. *Clinical Infectious Diseases*.

[2] Sultan HY, Boyle AA, Sheppard N (2012). Necrotising fasciitis. *British Medical Journal*.

[3] Frazee BW, Fee C, Lynn J (2008). Community-acquired necrotizing soft tissue infections: a review of 122 cases presenting to a single emergency department over 12 years. *Journal of Emergency Medicine*.

[4] Hussein Q, Ananya D (2013). Necrotizing soft tissue infections. *Critical Care Clinics*.

[5] Bosshardt TL, Henderson VJ, Organ CH (1996). Necrotizing soft-tissue infections. *Archives of Surgery*.

[6] Jinn W, Hwee L (2013). Necrotizing fasciitis: eight-year experience and literature review. *The Brazilian Journal of Infectious Diseases*.

[7] Golger A, Ching S, Goldsmith CH, Pennie RA, Bain JR (2007). Mortality in patients with necrotizing fasciitis. *Plastic and Reconstructive Surgery*.

[8] Smith-Slatas CL, Bourque M, Salazar JC (2006). *Clostridium septicum* infections in children: a case report and review of the literature. *Pediatrics*.

[9] Zacharias N, Velmahos GC, Salama A (2010). Diagnosis of necrotizing soft tissue infections by computed tomography. *Archives of Surgery*.

[10] Holland MJ (2009). Application of the laboratory risk indicator in necrotising fasciitis (LRINEC) score to patients in a tropical tertiary referral centre. *Anaesthesia and Intensive Care*.

[11] Yen Z-S, Wang H-P, Ma H-M, Chen S-C, Chen W-J (2002). Ultrasonographic screening of clinically-suspected necrotizing fasciitis. *Academic Emergency Medicine*.

[12] Oelze L, Wu S, Carnell J (2013). Emergency ultrasonography for the early diagnosis of necrotizing fasciitis: a case series from the ED. *The American Journal of Emergency Medicine*.

[13] Parenti GC, Marri C, Calandra G, Morisi C, Zabberoni W (2000). Necrotizingfascitis of soft tissues: diagnostic imaging findings and literature review. *Radiologia Medica*.

[14] Chau CLF, Griffith JF (2005). Musculoskeletal infections: ultrasound appearances. *Clinical Radiology*.

[15] Tunovic E, Gawaziuk J, Bzura T, Embil J, Esmail A, Logsetty S (2012). Necrotizing fasciitis: a six-year experience. *Journal of Burn Care and Research*.

[16] Young MH, Aronoff DM, Engleberg NC (2005). Necrotizing fasciitis: pathogenesis and treatment. *Expert Review of Anti-Infective Therapy*.

[17] Soh CR, Pietrobon R, Freiberger JJ (2012). Hyperbaric oxygen therapy in necrotising soft tissue infections: a study of patients in the United States Nationwide Inpatient Sample. *Intensive Care Medicine*.

